# Upper- and mid-mantle interaction between the Samoan plume and the Tonga–Kermadec slabs

**DOI:** 10.1038/ncomms10799

**Published:** 2016-02-29

**Authors:** Sung-Joon Chang, Ana M. G. Ferreira, Manuele Faccenda

**Affiliations:** 1 Division of Geology and Geophysics, Kangwon National University, Chuncheon, Gangwon-do 24341, South Korea; 2 Department of Earth Sciences, University College London, London WC1E 6BT, UK; 3 CERIS, Instituto Superior Técnico, Universidade de Lisboa, Av. Rovisco Pais 1, 1049-001 Lisboa, Portugal; 4 Dipartimento di Geoscienze, Università di Padova, 35131 Padova, Italy

## Abstract

Mantle plumes are thought to play a key role in transferring heat from the core–mantle boundary to the lithosphere, where it can significantly influence plate tectonics. On impinging on the lithosphere at spreading ridges or in intra-plate settings, mantle plumes may generate hotspots, large igneous provinces and hence considerable dynamic topography. However, the active role of mantle plumes on subducting slabs remains poorly understood. Here we show that the stagnation at 660 km and fastest trench retreat of the Tonga slab in Southwestern Pacific are consistent with an interaction with the Samoan plume and the Hikurangi plateau. Our findings are based on comparisons between 3D anisotropic tomography images and 3D petrological-thermo-mechanical models, which self-consistently explain several unique features of the Fiji–Tonga region. We identify four possible slip systems of bridgmanite in the lower mantle that reconcile the observed seismic anisotropy beneath the Tonga slab (*V*_SH_>*V*_SV_) with thermo-mechanical calculations.

## Abstract (web summary)

Plume-slab interaction and its surface expression remain poorly understood. Here, the authors compare 3-D anisotropic tomography and geodynamical models, and show that the stagnation and fastest trench retreat of the Tonga slab are consistent with an interaction with the Samoan plume and the Hikurangi plateau.

## Graphical abstract

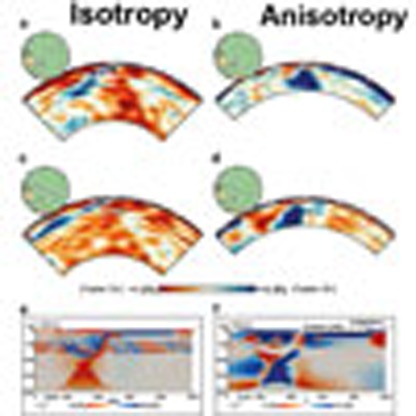

The Fiji–Tonga area in the Southwest Pacific shows many unique features. First, the Tonga slab is not only the fastest subducting slab on Earth, at a speed of 24 cm per year, but also accommodates the fastest back-arc opening within the Lau Back-arc Basin at a rate of 17 cm per year (ref. 1) (Fig. 1). Second, it shows intense seismicity, with the number of reported deep earthquakes in the transition zone being 10-fold larger than in any other subduction zone^2^. Third, anomalous Ocean Island Basalt (OIB) signatures such as very high ^3^He/^4^He ratios and latitudinal gradients in trace element and isotopic (Sr–Nd–Pb) enrichment are found in the northwestern corner of the Lau Basin and nearby regions (Fig. 1) (refs 3, 4, 5, 6, 7, 8, 9, 10), which appear to originate from the Samoan plume. A final distinctive feature is the strong upward deflection of the 660 km mantle discontinuity beneath the region^11^ that may indicate plume-related upwelling of hot materials from the lower mantle. Other studies^12^,^13^ also support high temperature in the upper mantle beneath the North Fiji Basin and the Lau Basin from geological, geochemical and geophysical evidence. Recent seismic tomographic models^14^,^15^ reveal a complex subduction slab morphology in the region, with a stagnant slab above the 660-km discontinuity in the northernmost Tonga region and a penetrating slab below the 660-km discontinuity further south, where the Hikurangi plateau is entrained into the subduction zone (Fig. 1). However, the spatial distribution, extent and direction of mantle flow from the Samoan plume and its effect on the slabs still remain enigmatic.

Here we suggest a self-consistent hypothesis to explain all the aforementioned phenomena by comparing results from three-dimensional (3D) anisotropic tomography and 3D geodynamic modelling. To constrain the region's dynamical processes, it is necessary to image not only the isotropic structure (for example, *Vs* and *Vp* in three dimensions, which do not give direct insight into mantle flow), but also the anisotropic structure, since anisotropy can be a proxy for deformation and the pattern of mantle flow. Because uncertainties in corrections for crustal structure can have a dramatic effect on the imaging of radial anisotropy^16^, we recently built a new global anisotropic tomography model that is able to overcome this problem by incorporating crustal thickness perturbations as model parameters along with a massive seismic data set (Methods)^17^,^18^.

## Results

### Seismic tomography

**Figure 2:**
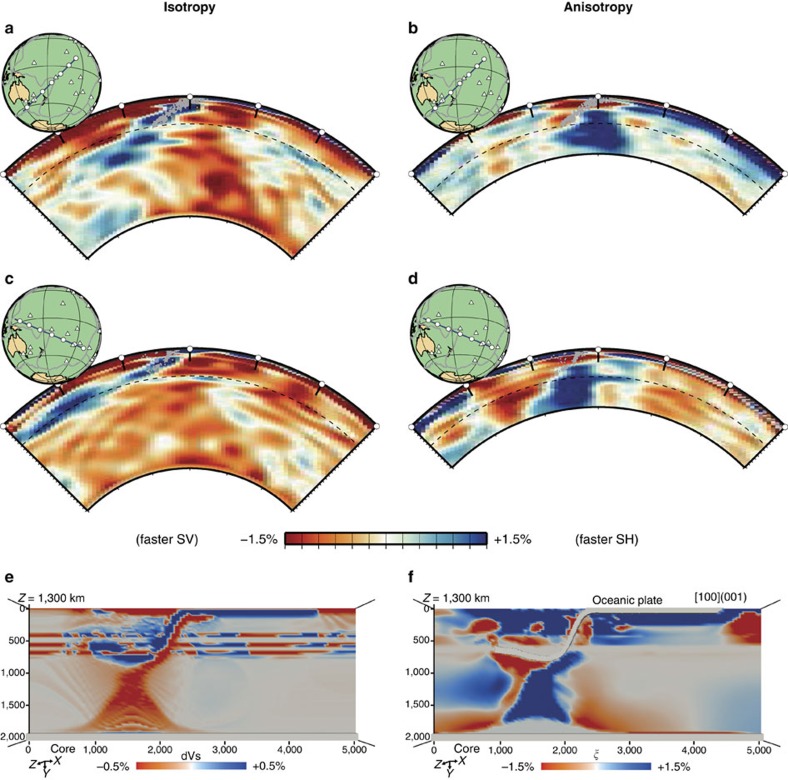
Cross sections of perturbations in Voigt average and anisotropic structure and corresponding geodynamic models. (**a**,**c**) Cross sections from the Voigt average model (
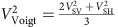
) in the direction of SW–NE and NW–SE, respectively. (**b**,**d**) Cross sections from the anisotropic model (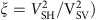 in the direction of SW–NE and NW–SE, respectively, down to 1,400 km depth, from where the resolution of the anisotropic structure is limited^18^. Focal depths from EHB data^58^ with an upper bound of 60 km are superimposed in the cross sections as grey circles. The mantle discontinuity at 660 km is indicated by black-dashed lines in cross sections. Hot spots are represented as triangles in the in-maps. (**e**,**f**) Isotropic dVs and radial anisotropy from the geodynamic modelling shown in Fig. 4d. The vertical cross-section is taken at *Z*=1,300 km. In **e** the anomalies are due to (1) variations of temperature and (2) topography of major mantle phase transitions occurring approximately at 410, 520 and 660 km depth. In **f** radial anisotropy in the lower mantle results from the strain-induced fabric of bridgmanite calculated with easy [100](001) system five times weaker than all other slip systems (Supplementary Fig. 8).

**Figure 3:**
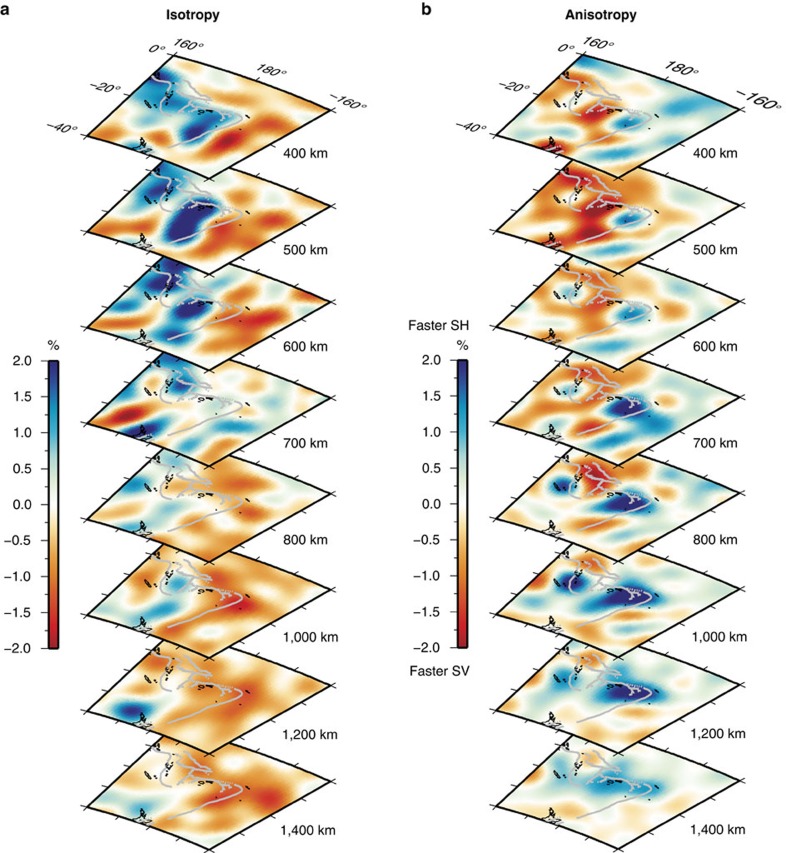
Depth slices of perturbations in Voigt average and anisotropic structure. (**a**,**b**) Depth slices from the Voigt average model (
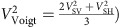
 and from the anisotropic model (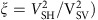 at depths of 400, 500, 600, 700, 800, 1,000, 1,200 and 1,400 km, respectively. Plate boundaries are indicated by grey lines.

Our isotropic structure model (Figs 2a,c and 3a; Supplementary Fig. 1a), shows the Kermadec and Tonga slabs as high-velocity anomalies (blue colour), while a large continuous low-velocity anomaly upwelling (red colour) from the core–mantle boundary reaches the Tonga slab in the uppermost lower mantle. This upwelling appears to originate from the location of a reported mega-sized ultra-low-velocity zone (ULVZ)^19^ in the large low shear-wave velocity province beneath the Pacific. The Tonga slab, which is in contact with the mantle plume upwelling, is stagnant in the mantle transition zone, while further south the Kermadec slab penetrates into the lower mantle (Supplementary Fig. 1a; Supplementary Movie 1), consistent with other recent studies^14^,^15^.

As for the anisotropic structure (Figs 2b,d and 3b; Supplementary Fig. 1b; Supplementary Movie 2), a faster SH velocity anomaly of ∼2% (blue colour) is observed behind the Tonga slab and reaches down to ∼1,400 km depth. Below this depth the resolution of the anisotropic structure is limited and hence the anomaly is not resolvable^18^. An anisotropy anomaly (*V*_SH_>*V*_SV_) in this region has been previously suggested^20^, but we have been able to better constrain its extent both vertically and laterally with our global tomography approach. Unlike previous interpretations of a thin anomaly (∼100 km) (ref. 20), we find the anomaly to be thick (∼1,000 km). In the transition zone (at ∼410–660-km depth) a region with faster SV velocity is observed near the slabs. Extensive resolution tests show that these observed features are robust (Supplementary Figs 2–4). We observe anomalies of faster SH velocity beneath other stagnant slabs as well^18^. For example, a faster SH anomaly reaches down to around 1,200 km beneath the Japan trench where no plume activity exists (Supplementary Fig. 5). Given the good resolution beneath the Japan trench, this may indicate that the faster SH velocity beneath the stagnant slab may be caused by subduction-induced shear deformation^21^, which is reproduced in the geodynamic modelling in Supplementary Fig. 6. However, the anomaly beneath the Tonga slab is deeper and stronger than in other regions (Supplementary Fig. 5), strongly suggesting a contribution of the Samoan plume to this anomaly.

### Geodynamic modelling

**Figure 4:**
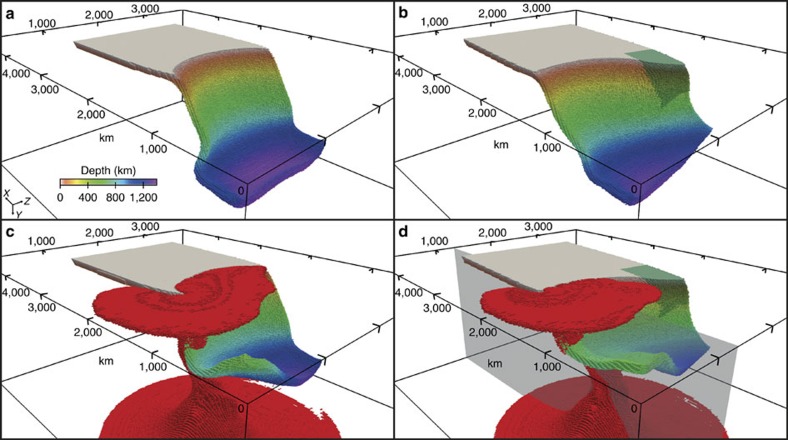
Role of the upwelling plume and subducting plateau on subduction dynamics. Comparison of four different models at 32 Myr characterized by: (**a**) oceanic plate only; (**b**) oceanic plate and plateau (transparent light green); (**c**) oceanic plate and plume (red); (**d**) oceanic plate, plateau and plume. The transparent grey plane in **d** indicates the position of the dVs and radial anisotropy cross sections displayed in Fig. 2e,f and Supplementary Fig. 8. The oceanic plate is coloured according to the depth of the material. To avoid colour-coding confusion, the core material at the bottom of the domain is not visualized.

**Figure 5:**
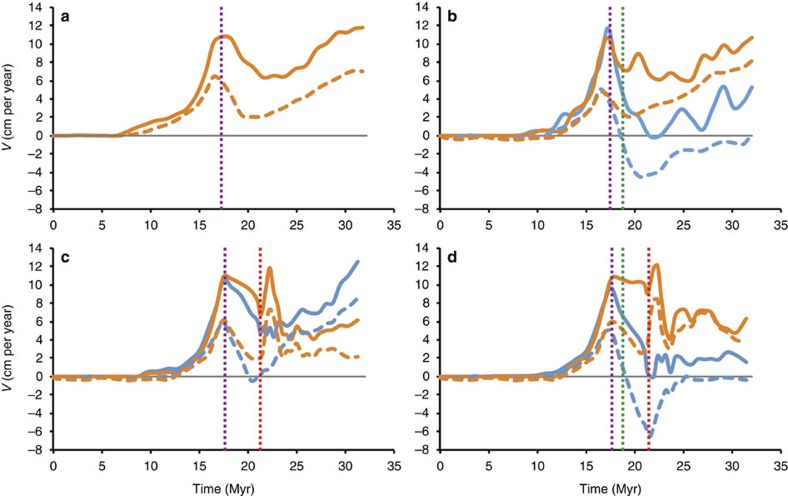
The trench (dashed line) and subduction (continuous line) velocities for all the four models and for the left (blue) and right (orange) plate margins. (**a**) oceanic plate only (velocities taken only at the centre of the symmetric plate); (**b**) oceanic plate and plateau; (**c**) oceanic plate and plume; (**d**) oceanic plate, plateau and plume. The purple, green and red lines mark the arrival of slab at the 660-km discontinuity (decreasing both velocities), the arrival of the plateau at the trench (causing trench advance and strong trench retreat on the opposite side of the trench, see Supplementary Fig. 7), and beginning of the interaction slab–plume in the transition zone (which increases shortly, and then slows down the velocities where it impinges, while accelerating the kinematics on the opposite side of the plate).

Geodynamic modelling offers a unique tool to investigate mantle dynamics during plume–slab interaction. Using the modelling strategy of ref. 21 (Methods), we find that both the recent tectonic evolution and the present-day seismological observations of the Tonga–Kermadec subduction zone can be reproduced when an upwelling plume hits the bottom of a subducting oceanic plate in the transition zone (Fig. 4; Supplementary Movies 3 and 4). We chose a relatively large plume, based on the extent (∼1,000 km) of the deflection of the 660-km discontinuity^11^ and the resolving power of our model, which is confirmed in another recent waveform tomography study^22^. On the one hand, the upwelling plume favours stagnation in the transition zone of the overlying slab segment (Fig. 4c,d) and increases trench retreat at the opposite side of the subduction system^23^ (Supplementary Fig. 7c). It is worth noting that slab stagnation is found also in models where the plume is upwelling beneath the centre of the plate. On the other hand, the entrainment of the Hikurangi plateau arrests trench motion and decreases the rate of subduction on the Kermadec slab^23^,^24^,^25^, while promoting fast (up to >9 cm per year) trench retreat on the Tonga slab (Figs 4b,d and 5; Supplementary Fig. 7 for the trench position with time). The fast trench rollback, in turn, increases the subduction velocity and the tendency of the slab to stagnate in the transition zone by decreasing the slab dip angle^26^, and induces strong toroidal mantle flow patterns around the edge of the Tonga slab, which bring hot plume materials into the mantle wedge. Near the edge of the subduction zone, the upper mantle and upper transition zone are characterized by mostly positive and negative radial anisotropy, respectively, due to toroidal flow-related deformation (Fig. 2f). In the uppermost lower mantle, a broad and thick radial anisotropy anomaly is associated with the hot plume due to plume–plate interaction. This anomaly is juxtaposed with a ribbon-like radial anisotropy anomaly located beneath the flat slab segment and which is due to subduction-induced deformation (for example, refs 21, 27). By systematically investigating several potential bridgmanite fabrics (Methods), a positive radial anisotropy (*V*_SH_>*V*_SV_) anomaly is associated with the plume when the dominant slip system is either [100](010), [100](001), 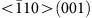 or <110>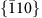 (Fig. 2f; Supplementary Fig. 8). These slip systems are consistent with those identified in high-pressure deformational experiments on bridgmanite^28^,^29^. Consequently, the results from geodynamic modelling support our view that the unusually large and strong upwelling seen in the seismological model may account for the intense deformation beneath the Tonga slab, hampering the penetration of the Tonga slab into the lower mantle, unlike the Kermadec slab.

## Discussion

After colliding with the Tonga slab, the Samoan plume seems to change its upward direction to parallel to the slab (Figs 2a,c and 4d). In the upper mantle, plume materials migrate into the mantle wedge around the northern tip of the Tonga slab^30^ (white arrow in Supplementary Fig. 1c), consistent with our 3D thermal–mechanical simulations and with results from laboratory experiments^31^. Corresponding anisotropy shows faster SH than SV velocity (Supplementary Fig. 1d), which may indicate the horizontal flow in the upper mantle assuming the presence of A-type fabric in olivine. The plume material's migration is responsible for OIB signatures in the Rochambeau Rift in the northwestern corner of the Lau Basin and nearby regions^3^,^4^,^5^,^6^,^7^,^8^,^9^,^10^, being pervasive throughout the Lau and North Fiji Basins.

**Figure 6:**
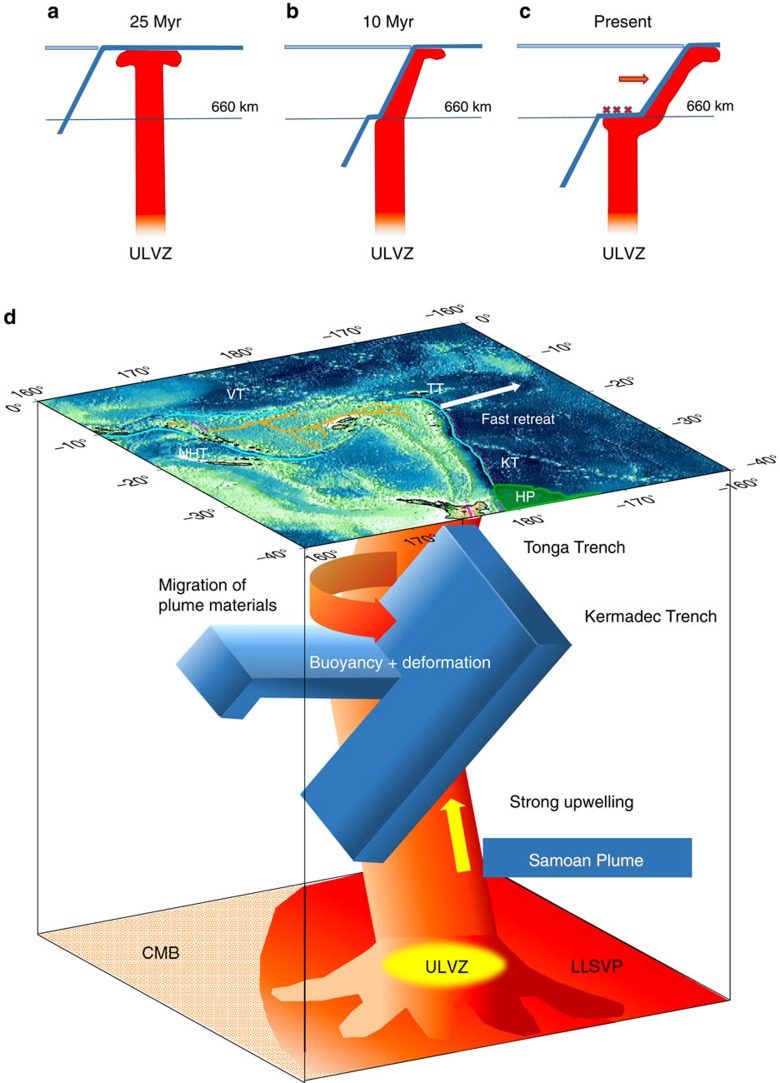
Illustrated time history of the plume–slab interaction and a cartoon for summarizing current features. (**a**) The Samoan plume is generated at the Mega ULVZ^19^ at the core–mantle boundary and is ascending to the surface. (**b**) The Samoan plume collided with the Tonga slab at the transition zone at about 10 Myr. (**c**) The upward stress by the collision has caused the stagnant slab and intense seismicity (cross marks), which is further enhanced by fast slab retreat (red arrow) due to the subduction of the Hikurangi plateau. (**d**) A schematic diagram illustrating the slab–plume interaction beneath the Tonga–Kermadec arc. Cyan lines on the surface show trenches, as shown in Fig. 1. HP, Hikurangi Plateau; KT, Kermadec Trench; NHT, New Hebrides Trench; TT, Tonga Trench; VT, Vitiaz Trench. The Samoan plume originates from a Mega ULVZ at the core–mantle boundary (CMB). The buoyancy caused by large stress from the plume at the bottom of the Tonga slab may contribute to the slab stagnation within the mantle transition zone, while the Kermadec slab is penetrating into the lower mantle directly. At the northern end of the Tonga slab, plume materials migrate into the mantle wedge, facilitated by strong toroidal flow around the slab edge induced by fast slab retreat.

In Fig. 6, we summarize our interpretation of the main features of the mantle flow around the Tonga–Kermadec slabs and their interaction with the Samoan plume. A mega ULVZ at the core–mantle boundary seems to generate an unusually large mantle plume, which ascends through the transition zone (Fig. 6a). The plume collided with the Tonga slab at the bottom of the mantle transition zone at ∼10 Myr, since the likely location of the Tonga slab at that time^15^ coincides with that of the Samoan plume in our model. This collision caused intense deformation and buoyancy (Fig. 6b), contributing to slab stagnation and probably leading to the significant observed seismicity. During the past 10 Myr, the length of the stagnant slab is thought to have increased to about 700–800 km (refs 14, 15), which is consistent with a probable subduction rate of 7.2–14.2 cm per year (ref. 15), considering slab buckling and compression (Fig. 6c). The plume–slab collision time of 10 Myr is consistent with the beginning of the fast slab retreat^32^, which has caused the migration of plume materials into the mantle wedge, around the edge of the Tonga slab (Fig. 6d). However, the fast retreat of the northern Tonga–Kermadec trench margin is mostly ascribable to the entrainment at depth of the Hikurangi plateau, which, together with the adjacent Chatham Rise–plate boundary interaction, provides a regional impediment to subduction of the Pacific plate.

Our high-resolution isotropic and radially anisotropic models interpreted together with 3D petrological-thermo-mechanical numerical simulations provide the clearest picture so far of how mantle plumes can interact with subducting slabs from the mid mantle to the Earth's surface. Our detection of the large and very active Samoan plume ties together many different previous observations^1^,^2^,^3^,^4^,^5^,^6^,^7^,^8^,^9^,^10^,^11^,^12^,^13^,^14^,^15^,^27^,^30^,^31^,^32^ providing observational evidence that mantle plumes can be big and strong, able to contribute to slab stagnation and possibly to intense deep seismicity, and the coupled plume-fast slab retreat effect can further enhance slab stagnation and deformation. Since retreating slabs seem to dominate the Earth's subduction system^1^, significant interactions between fast retreating slabs and upwellings may be more common on Earth than previously thought^23^. Up to now, such interactions may have remained largely undetected due to limited seismic resolution and to the lack of fully concerted seismic and geodynamical interpretations. Our models open the way to hunt for possible hidden plumes beneath other slabs stagnating in the mid-mantle, to predict their geochemical and subduction kinematics signature in the geological record and to unravel their role on slab deformation, with potential strong implications in terms of the nature of material exchange between the Earth's upper and lower mantle. Our unique approach combining for the first time constraints from seismic tomography and the dynamical modelling of anisotropy in a systematic way also promises to reveal unprecedented details about the evolution of other regional settings.

## Methods

### Radially anisotropic mantle model

The novelty of our strategy is to use a massive and diverse data set, and to incorporate Moho perturbations to the inversions to address crustal effects consistently^17^,^18^ . We use the same inversion scheme as in ref. 16, with body-wave travel times added to the modelling using the theoretical developments of Woodhouse and his colleagues^33^,^34^. The models are parameterized horizontally using spherical harmonic basis functions expanded up to degree 35 (nominal lateral resolution of ∼600 km), and 21 spline functions are used for variations in the radial direction (see, for example, Fig. 4 in ref. 35). Horizontal norm damping is applied for regularization, and we adopt PREM^36^ as the reference model. We applied 1.3 times more damping to the anisotropic parameters than to the isotropic parameters to avoid over-interpretation, because of weaker sensitivity of the data to the anisotropic structure and consequently poorer resolution. Since we do not invert for seismic velocity in the crust, before the inversions we correct all the data using crustal corrections from the model CRUST2.0 (ref. 37). Therefore, our strategy to deal with the crust is a hybrid one; first, we carry out crustal corrections using CRUST2.0 taking into account crustal velocity and crustal thickness. Then, in our inversions, we estimate crustal thickness perturbations from CRUST2.0 using our data sets, which include group velocity data. The crustal thickness perturbations are estimated simultaneously to variations in 3D shear-wave velocity and radial anisotropy in the whole mantle.

Isotropic and anisotropic parameters are represented respectively as follows:


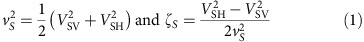


To keep the problem tractable, we scale perturbations of *V*_*P*_ and density to perturbations of *V*_*S*_ using the scaling relations 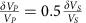 and 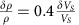, respectively^38^,^39^.

Since the model parameters in this study consist of perturbations of isotropic *S* velocity, *S* radial anisotropy and crustal thickness, the inverse problem can be written as follows:


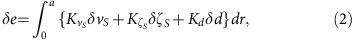


where *δe* is a measure of misfit between the data and theoretical calculations for a given reference model, *r* is the Earth's radius parameter, *a* is the total radius at the Earth's surface, and *δν*_*S*_, *δζ*_*S*_ and *δd* indicate perturbations of isotropic *S* velocity, *S* radial anisotropy and discontinuities with respect to the reference model. 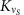, 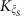 and *K*_*d*_ are depth sensitivity kernels with respect to isotropic *S* velocity, *S* anisotropy and discontinuities, respectively. For comparison with other models, we convert the parameters to the Voigt average isotropy (
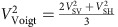
) and radial anisotropy (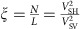) in the main paper.

Depth sensitivity kernels with respect to phase velocities are calculated using the approach of ref. 40, while the formulation of ref. 41 is used to compute group velocity kernels. Sensitivity kernels with respect to crustal thickness are calculated by following ref. 42 for phase velocity data and numerical differentiation for group velocity data. The great circle approximation is used to linearly relate the various datasets to Earth's structure^43^.

### 3D petrological-thermo-mechanical modelling

We used I3MG, a 3D geodynamic framework based on a mixed Eulerian–Lagrangian finite difference scheme^44^. The numerical domain (*X*–*Y*–*Z*: 5,000 × 2,000 × 4,000 km) is discretized with 245 × 197 × 101 Eulerian nodes, while the material properties are defined on and advected by ∼40 millions Lagrangian particles. The initial model set-up is characterized by a 3,300 × 80 × 2,000 km oceanic plate connected to a gently dipping, 335 km long slab, which drives subduction self-consistently (Supplementary Fig. 9a; Supplementary Table 1). Depending on the type of model, a relatively buoyant (2,950 kg m^−3^) 1,000 × 30 × 500 km plateau is prescribed on the left side of the plate, and a thermally buoyant plume is defined at the bottom of the mantle through a two-dimensional Gaussian function centered at (*X*=1,600 km; *Z*=1,500 km; that is, on the front right side of the plate) with height and width of 500 km (plume volume is 7.854 × 10^8^ km^3^, ∼2% of the domain volume). A 100-km thick, low viscosity, high-density layer at the bottom of the computational domain simulates the liquid core. Free slip is imposed on all boundaries.

The shallow thermal structure is calculated according to the half-space cooling model^45^ down to 90 km, then a constant thermal gradient of 0.5 K km^−1^ is assigned. The 70-Myr-old oceanic plate is juxtaposed with a 20-Myr-old mantle and a weak background crust, which produce moderate lateral friction on the plate. The plume has an initial constant temperature of 2,055 K, which is also the initial temperature at the simulated core–mantle boundary (*Y*=1,900 km). Consequently, the thermally induced buoyancy of the plume decreases towards such boundary.

A composite visco-plastic rheology based on deformation invariants has been used for the oceanic plate crust, as well as for the mantle and the overlying 30 km thick background crust surrounding the oceanic plate. The effective viscosity *η*_eff_ is the result of combined dislocation and diffusion creep mechanisms:


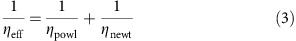


Power-law creep is given by:


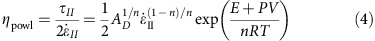


where *A*_*D*_, *E*, *V*, *n* are experimentally determined flow-law parameters and *τ*_II_ is the second invariant of the deviatoric stress tensor. For simplicity, the flow law of Dry Olivine^46^ is used for the whole mantle (Supplementary Table 1).

At low deviatoric stresses, thermally activated diffusion becomes the dominant creep mechanism. Following ref. 45, transition from dislocation to diffusion creep is prescribed at a given deviatoric stress, *τ*_II_trans_, implying that:


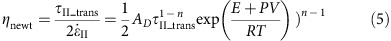



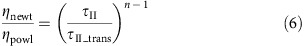


A low-transition stress value favouring dislocation creep is used for the upper mantle and transition zone, while for the lower mantle and the plume *τ*_II_trans_=30 MPa. Such rheological change across the transition zone—lower mantle boundary is implemented by varying *τ*_II_trans_ as the density of the mantle Lagrangian particles cross the arbitrary value of 4,150 kg m^−3^.

The strength of the material is limited by:


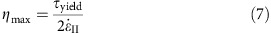


*τ*_yield_ is determined with a plastic Drucker–Prager criterion^46^:


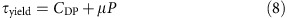


where *C*_DP_*=C*cos *φ* and *μ=*sin *φ* are the cohesion and coefficient of friction, *φ* is the friction angle.

The oceanic plate is made of a strong, 20-km-thick core with constant viscosity (10^24^ Pa·s) embedded in between two weaker layers 30-km thick. The lower layer has a constant viscosity of 3 × 10^22^ Pa·s, while the crust is visco-plastic. In such layer, brittle weakening is implemented as (Supplementary Table 1):


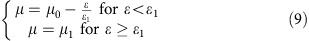


where *μ*_0_ and *μ*_1_ are the coefficient of friction at zero deformation and at strain *ɛ*_1_. Brittle weakening is needed to prevent subduction on the side of the slab and to ensure lubrication at the plates contact after bending-related deformation. Note also that because of the relatively low resolution, very low values of *μ*_0_, *μ*_1_ and *ɛ*_1_ are needed to ensure an efficient lubrication on the top boundary of the subducting slab. Although simplified, such layered rheological structure captures the essential characteristic of the lithosphere yielding profile (for example, ref. 47) producing realistic subduction patterns while preventing slab breakoff^48^,^49^,^50^.

The dynamics of subduction through the mid mantle is affected by several phase transitions that may favour or hinder the sinking of the slab/upwelling of the plume via buoyancy forces. These phase transitions are included by using *P*–*T*-dependent density and enthalpy maps generated with PERPLE_X^51^ for a pyrolitic mantle composition (Supplementary Fig. 9b).

### Mantle fabric modelling

The method for computing the lattice preferred orientation (LPO) of mantle polycrystalline aggregates is accurately described in refs 21, 50. The Lagrangian aggregates are homogeneously distributed (initial spacing is 50 × 30 × 50 km) within the computational domain and are passively advected by means of the Eulerian velocity field obtained by the macro-flow modelling. At each time-step, the fabric development of each aggregate is calculated according to the Eulerian velocity gradient field using D-Rex^52^, modified to account for non-steady-state deformation and deformation history^50^,^53^, combined diffusion–dislocation creep mechanisms and strain-induced LPO of mid-mantle aggregates^21^.

The modal abundances of the aggregates composed by 1,000 crystals reflect a pyrolitic mantle composition (Wd:Grt=60:40 for the upper transition zone, 410–520 km; Rw:Grt=60:40 for the lower transition zone, 520–660 km; Brd:MgO=80:20 for the lower mantle, 660–1,900 km), with the exception of the upper mantle where a more appropriate harzburgitic composition is chosen (Ol:Ens=70:30, 0–410 km) (ref. 54). Phase transition of the whole crystal aggregate is set to occur at arbitrary density crossovers that are assumed to represent the (sharp) boundary between two different rock types (Ol:Ens→Wd:Grt=3,650 kg m^−3^; Wd:Grt→Rw:Grt=3,870 kg m^−3^; Rw:Grt→Brd:MgO=4,150 kg m^−3^). Although part of the LPO can be inherited by aggregates during phase transformation^55^, when a given density boundary is crossed, the composition of the crystal aggregate is changed and its LPO is reset by randomizing the crystal orientation.

Fabric development is computed only for the fraction of viscous deformation accommodated by dislocation creep and only for phases such as olivine, enstatite, wadsleyite and bridgmanite, which display significant single-crystal visco-elastic anisotropy. Conversely, because aggregates of cubic phases such as ringwoodite, garnet and MgO-periclase are mostly isotropic in the mid mantle^56^, their crystal orientation is maintained random through the model run. As a result, the lower transition zone will appear as isotropic. The strain-induced LPOs of olivine, enstatite and wadsleyite are obtained by comparison with available experimental data (Supplementary Table 2). In contrast, very little is known about the mechanical properties and deformation mechanisms of bridgmanite at lower mantle *P*–*T* conditions. Nevertheless, several potential slip systems have been identified through *ab initio* simulations^57^ and high-pressure, low-strain deformational experiments^28^,^29^. Dominant slip systems in the uppermost lower mantle appear to be [100](010), [100](001), [010](100), [010](001), [001](100), [001](010), [001]{10}, <10>(001), <110>{110}. Consequently, we have tested different lower mantle LPOs. To understand the pattern of radial anisotropy associated with a given dominant slip system, most fabrics are characterized by an easy slip system much weaker than the others (Supplementary Fig. 10). An additional fabric has been obtained with normalized critical resolved shear stresses from static (0 K) *ab initio* atomic scale modelling run at pressure typical of the uppermost lower mantle^56^,^57^ (Supplementary Table 2). The different radial anisotropy patterns in the lower mantle resulting from these fabrics are reported in Supplementary Fig. 8.

The elastic properties of the aggregates are obtained by Voigt-averaging the crystal elastic properties (scaled by the local *P*–*T* conditions through *P*–*T* derivatives of the elastic moduli^21^) according to their volume and orientation. Interpolating the elastic moduli from the Lagrangian aggregates throughout the model domain allows us to calculate, for example, the radial anisotropy and isotropic *Vp* and *Vs* anywhere in the model (Fig. 2e,f; Supplementary Figs 8 and 9c).

## Additional information

**How to cite this article**: Chang, S. J. *et al.* Upper- and mid-mantle interaction between the Samoan plume and the Tonga–Kermadec slabs. *Nat. Commun.* 7:10799 doi: 10.1038/ncomms10799 (2016).

## Supplementary Material

Supplementary Information Supplementary Figures 1-10, Supplementary Tables 1-2 and Supplementary References.

Supplementary Movie 1 Three-dimensional isosurfaces from the Voigt average models from 60 km to 2800 km beneath the study region. Red and blue isosurfaces indicate -1 and +1 % perturbations, respectively. Cyan lines on the surface show trenches.

Supplementary Movie 2 Three-dimensional isosurfaces from the anisotropic models from 60 km to 2800 km beneath the study region. Red and blue isosurfaces indicate -1 and +1 % perturbations, respectively. Cyan lines on the surface show trenches.

Supplementary Movie 3 Self-consistent evolution of a numerical model characterized by a subducting oceanic plate with a relatively buoyant plateau and upwelling mantle plume (same as in Fig. 4d). Color scale as in Fig. 4 in the main text. View is toward the X axis.

Supplementary Movie 4 Self-consistent evolution of a numerical model characterized by a subducting oceanic plate with a relatively buoyant plateau and upwelling mantle plume (same as in Fig. 4d). Color scale as in Fig. 4 in the main text. View is from the left side of the numerical domain.

## Figures and Tables

**Figure 1:**
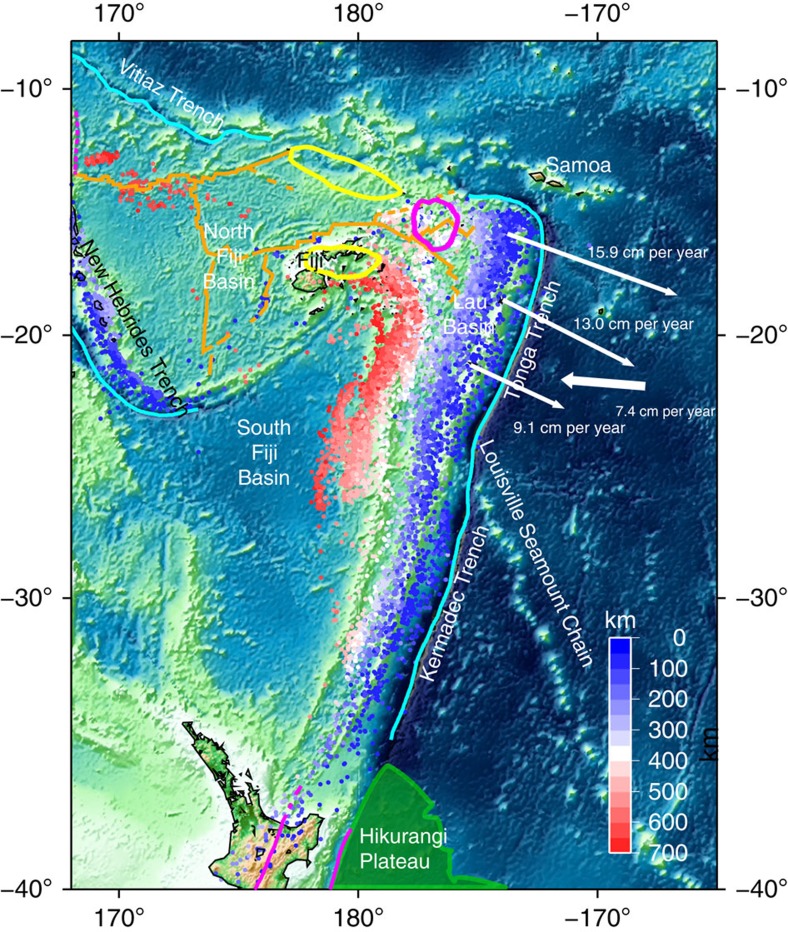
Bathymetry around the Tonga–Kermadec trench along with the region's seismicity. Seismicity with depth deeper than 60 km is obtained from Engdahl *et al.*^58^ and indicated by coloured circles according to the focal depth. Plate boundaries are depicted by solid lines: orange and cyan lines represent ridges and trenches, respectively. Magenta lines indicate transform faults. Regions where Ocean Island Basalts (OIB; high He^3^/He^4^ ratio) with a signature of the Samoan plume are found^3^,^4^,^5^ are indicated by enclosed solid magenta lines. Other possible signatures of the Samoan plume are found in the regions represented by yellow enclosed lines^6^,^7^,^8^,^9^,^10^. Relative velocity of the Pacific plate to the Australian plate is depicted by the thick white arrow based on MORVEL by DeMets *et al.*^59^ and velocities from GPS stations in an Australia-fixed reference frame are indicated by thin white arrows^60^.
