# Supplementary material for: Upper- and mid-mantle interaction between the Samoan plume and the Tonga–Kermadec slabs
Source: Nat Commun. 2016 Feb 29;7:10799. doi: 10.1038/ncomms10799 (PMC4773510; doi:10.1038/ncomms10799)
Supplement: Supplementary Information — Supplementary Figures 1-10, Supplementary Tables 1-2 and Supplementary References. [file ncomms10799-s1.pdf]

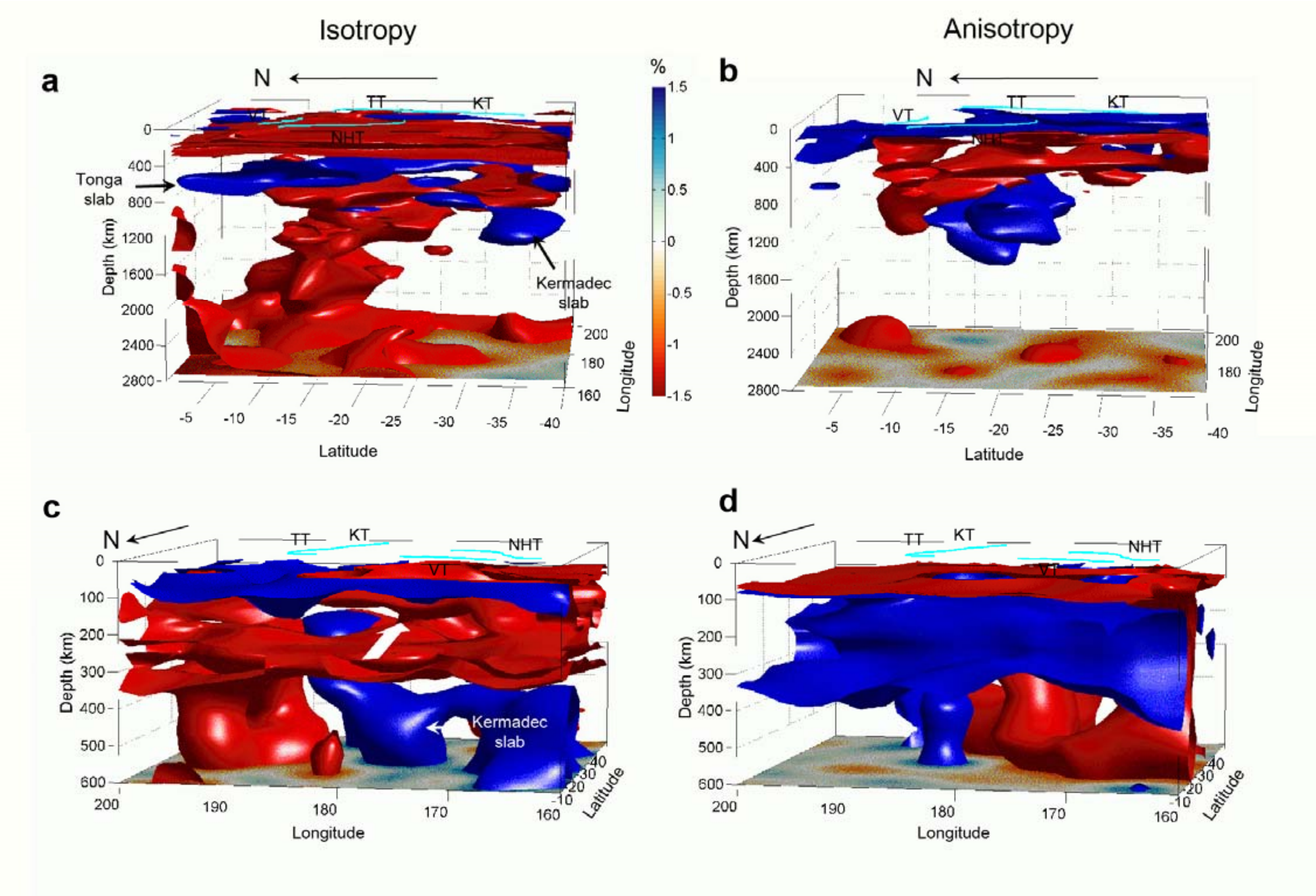

Supplementary Fig. 1:

Three-dimensional isosurfaces from the Voigt average (left) and anisotropic (right) models. Red and blue isosurfaces indicate -1 and +1 % perturbations. Cyan lines on the surface show trenches as shown in Fig. 1 in the main text. KT: Kermadec Trench; NHT: New Hebrides Trench; TT: Tonga Trench; VT: Vitiaz Trench. (**a,b**) 3D rendering of the Voigt average and anisotropic models for the depth range from 60 km to 2800 km beneath the study region. In (**a**) a big mantle plume (red) originating from a mega ULVZ at the core-mantle boundary reaches up to the surface. In **b** anisotropy with fast *SH* velocity (blue) is shown in a similar morphology to the mantle plume in **a**. (**c,d**) 3D rendering of the Voigt average and anisotropic models for the depth range from 60 km to 600 km, which are exaggerated vertically. In **c** the mantle plume (red) is intruding into the mantle wedge (white arrow) around the northern end of the Tonga slab. In **d** anisotropy with faster *SH* velocity (blue) follows the path of the mantle plume in **c**, suggesting horizontal flow based on experiments with olivine.

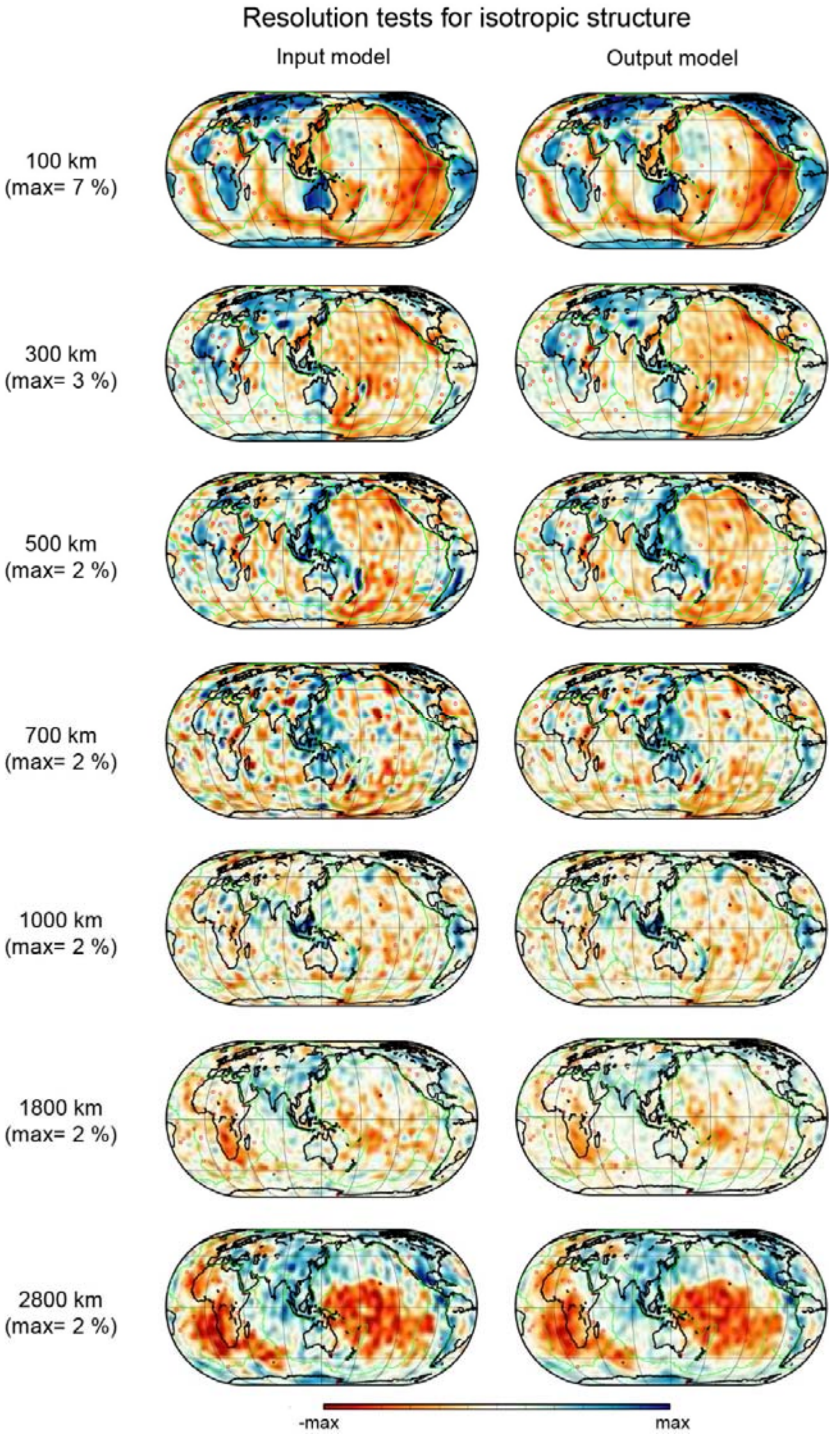

Supplementary Fig. 2:

Resolution tests with realistic structure for the isotropic model. The input model (left) is the isotropic part of the SGLOBE-rani model and resulting model is shown at various depths (right).

(right).

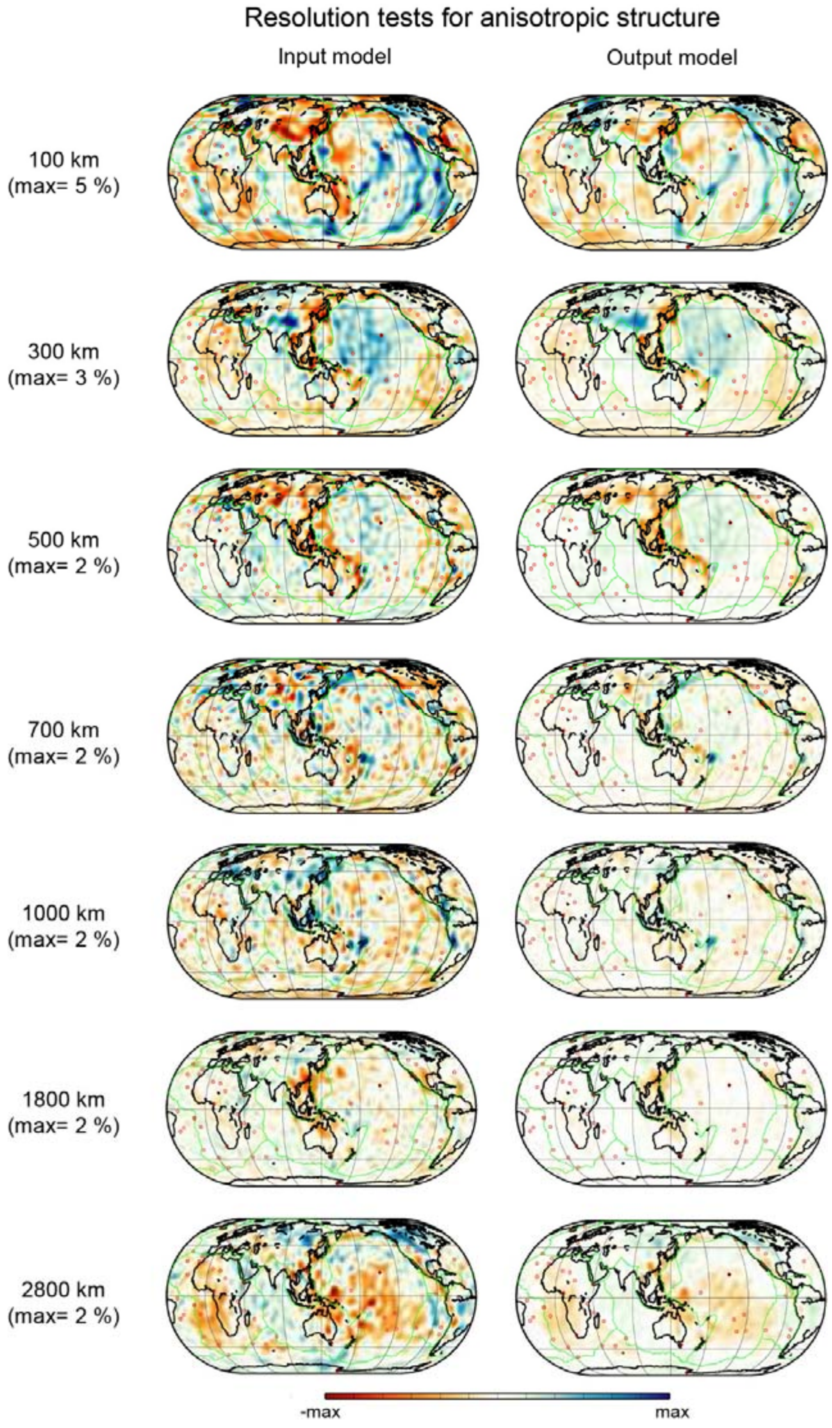

Supplementary Fig. 3:

Resolution tests with realistic structure for the anisotropic model. The input model (left) is the anisotropic part of the SGLOBE-rani model and resulting model is shown at various depths (right).

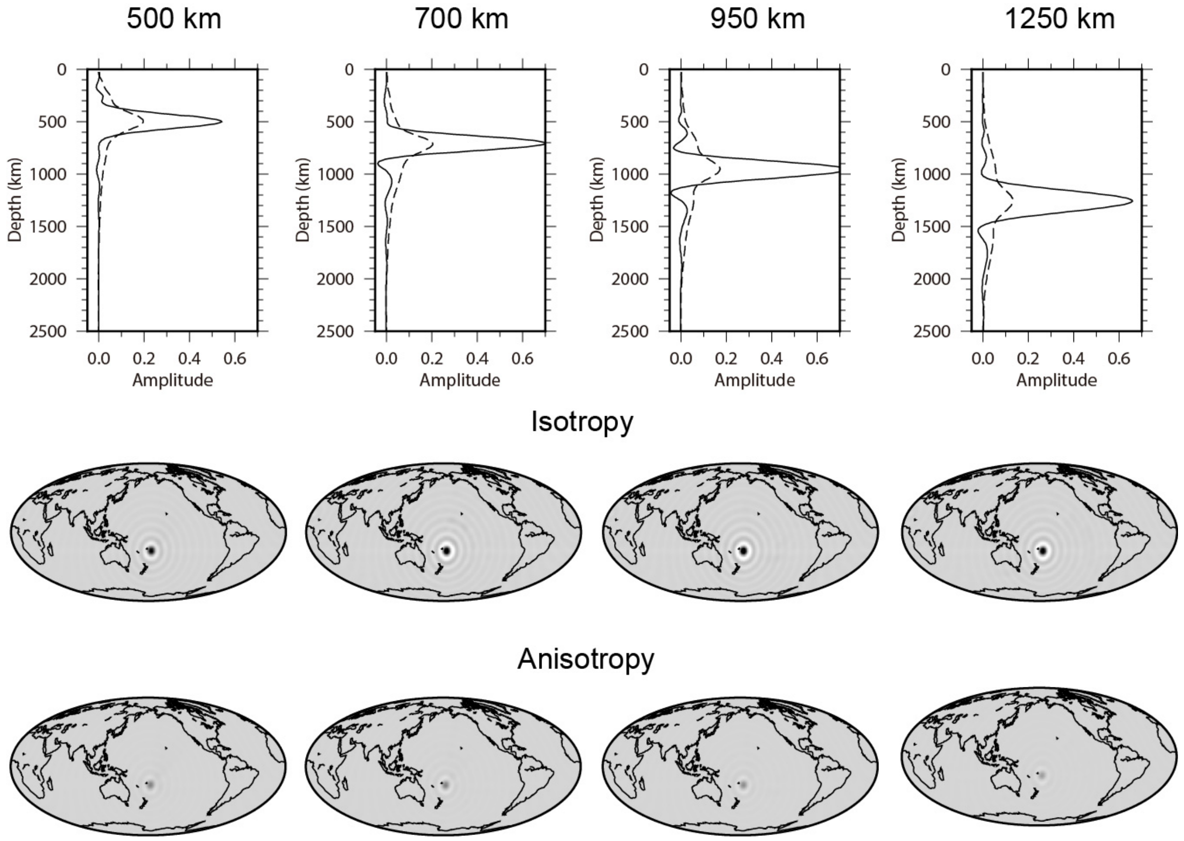

Supplementary Fig. 4:

Backus-Gilbert resolution kernels for the Fiji-Tonga area. They are calculated at 500, 700, 950, and 1250 km depth from left to right. The first row contains the radial dependence of the kernels. Solid and dashed lines represent kernels for isotropy and radial anisotropy, respectively. The second and third rows depict map views of kernels for isotropic and anisotropic structure, respectively.

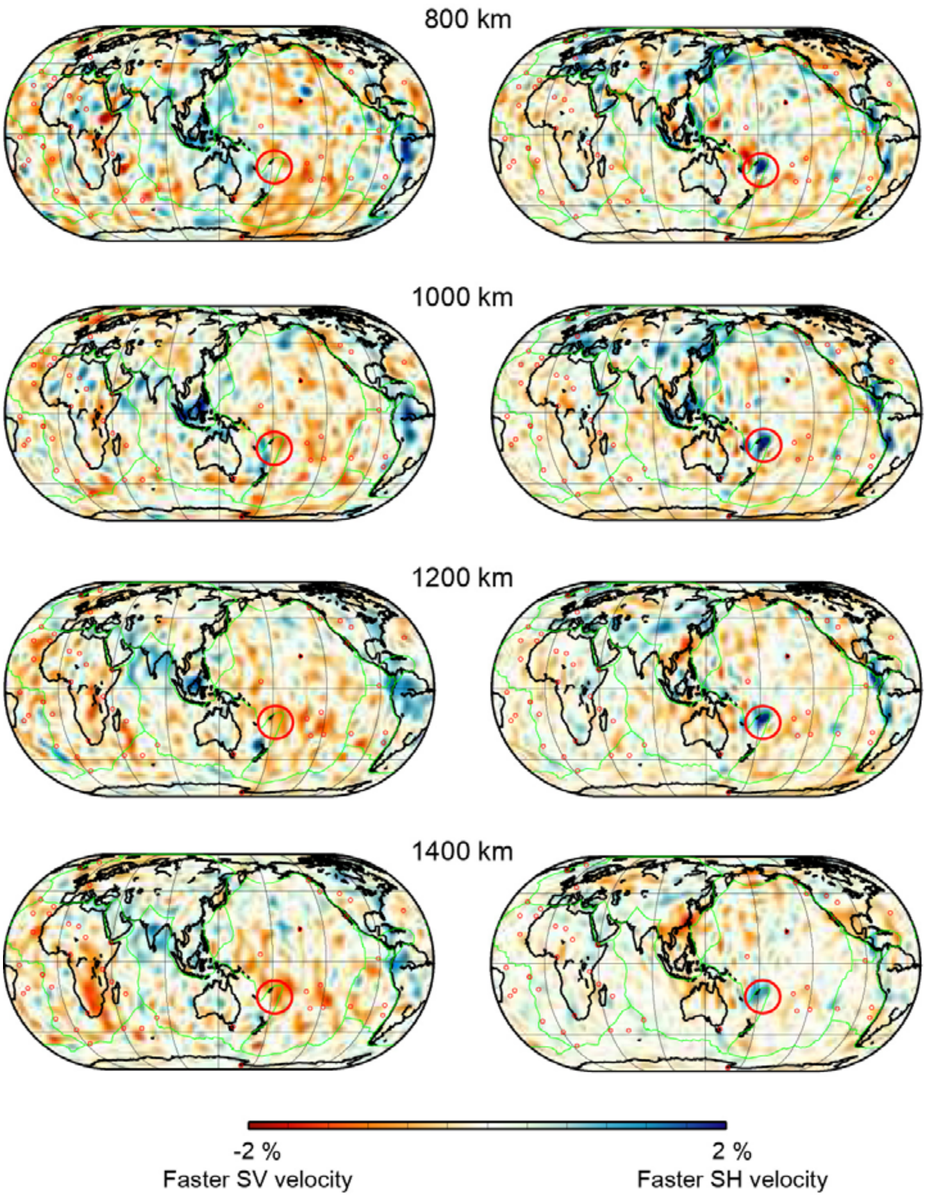

Supplementary Fig. 5:

Depth slices of perturbations of isotropy (left) and radial anisotropy (right). The strong Tonga anisotropic anomaly persists from the transition zone to 1400 km depth, which is a unique feature in the lower mantle.

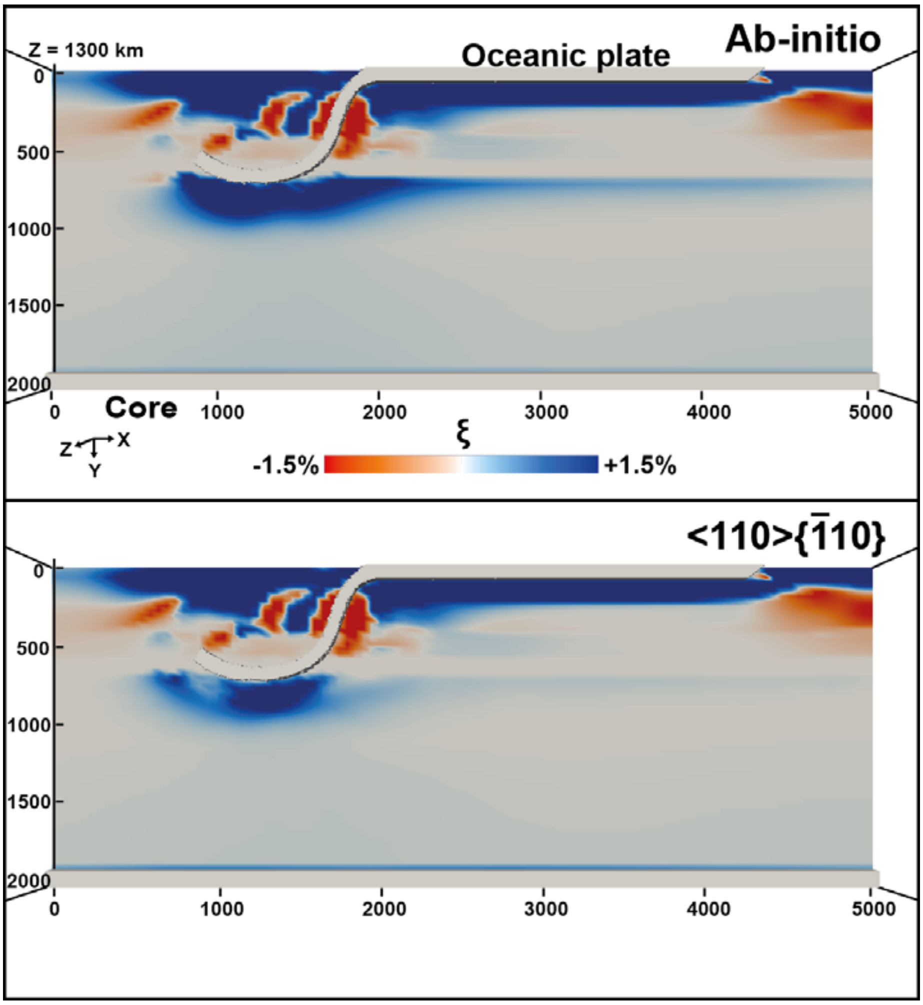

Supplementary Fig. 6:

Perturbations in radial anisotropy at Z = 1300 km. They are computed for the whole mantle in case of slab stagnation with no plume. The bridgmanite fabrics chosen in these two examples yield positive radial anisotropy below the slab at depths ≤ 1000 km. Differently from the model in Fig. 4a, the background geotherm is set to 1 Ma, which produces very small friction on the subducting plate, leading to fast trench retreat and slab stagnation[1].

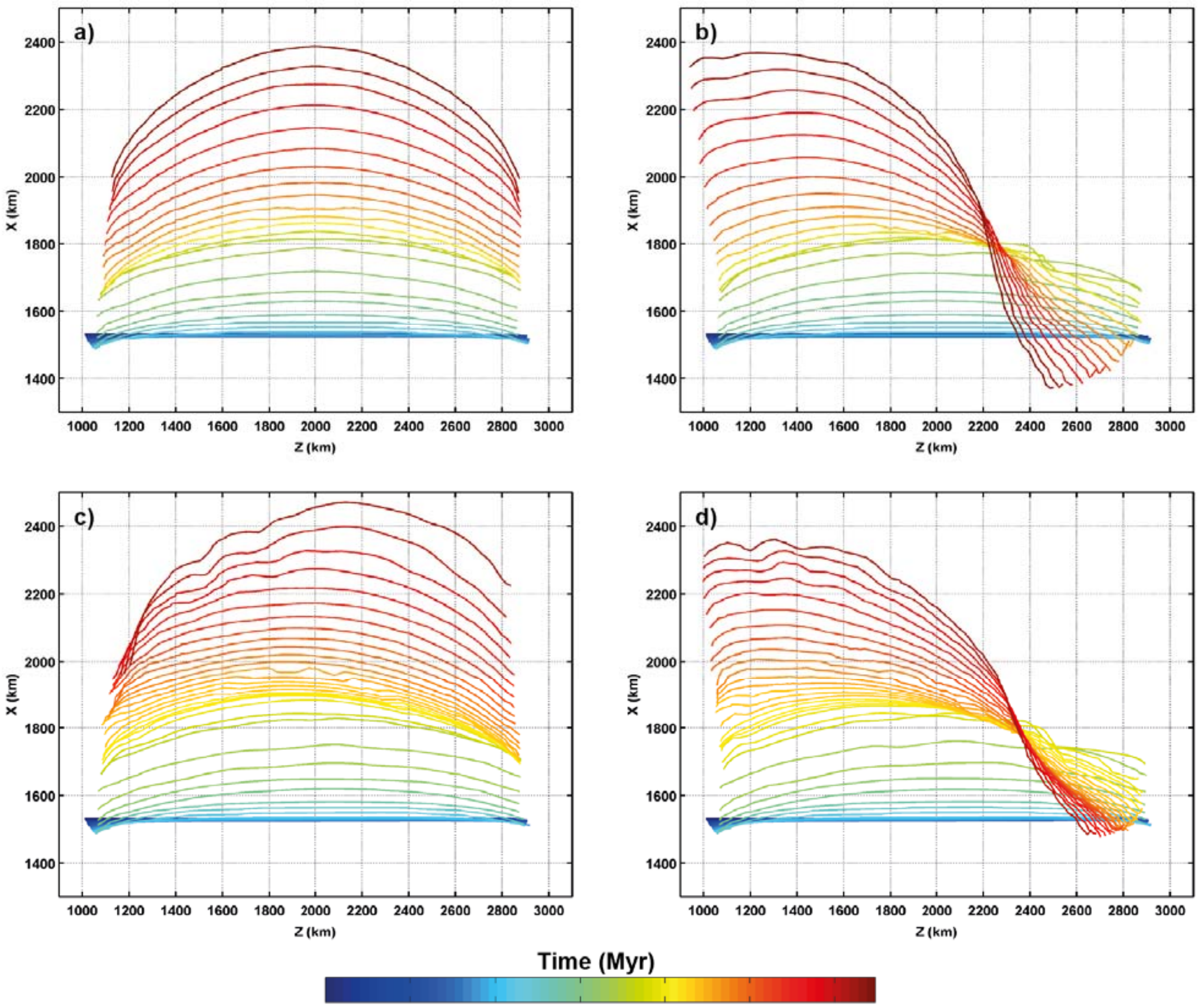

Supplementary Fig. 7:

The trench position of the four models. The trench position is depicted with time for the four models in Fig. 4.

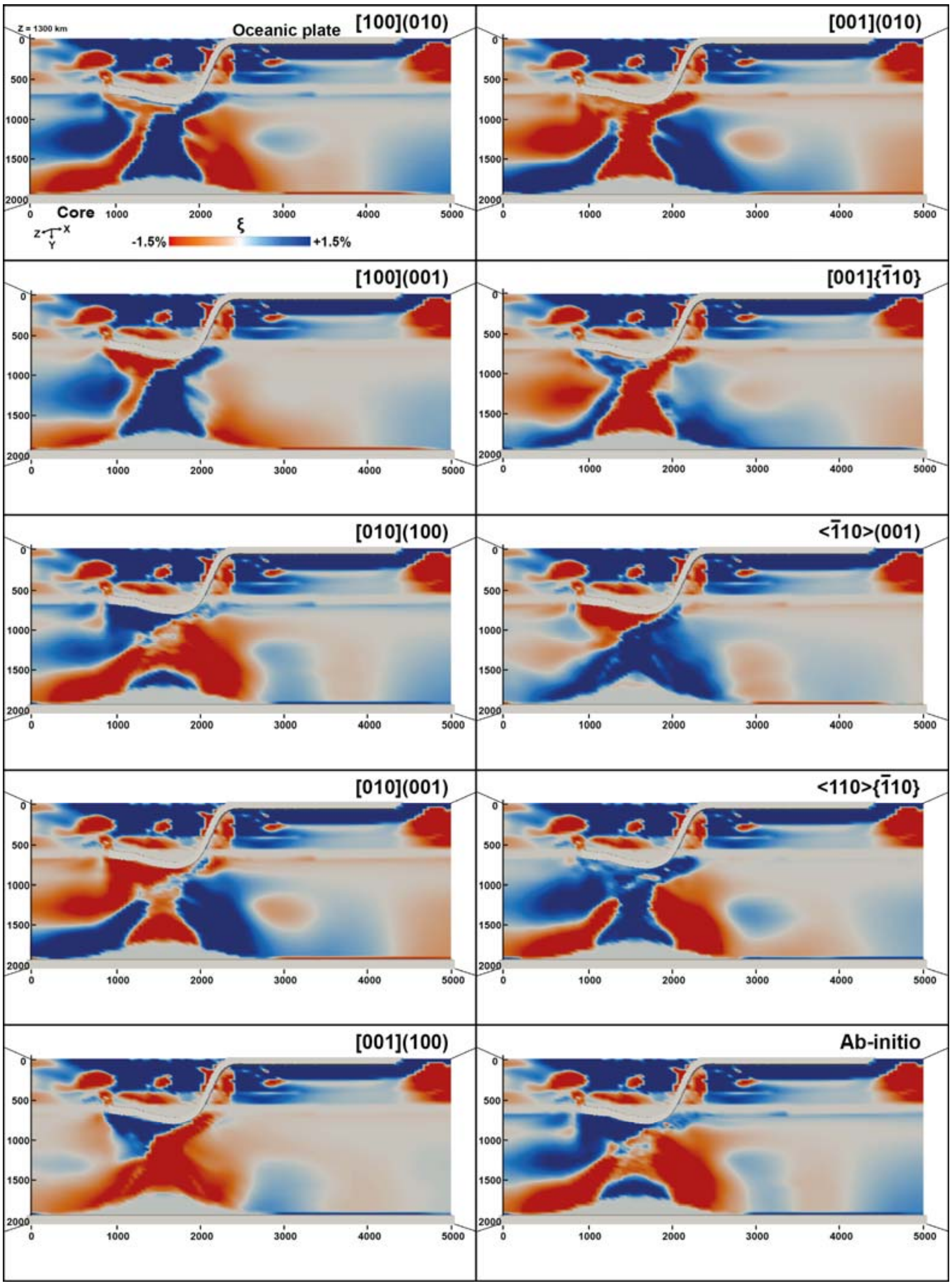

Supplementary Fig. 8:

Perturbations in radial anisotropy ξ at Z = 1300 km. They are computed for the whole mantle as a function of the different bridgmanite lattice-preferred orientations (LPOs) reported in Supplementary Fig. 10. The oceanic plate and the core are isotropic.

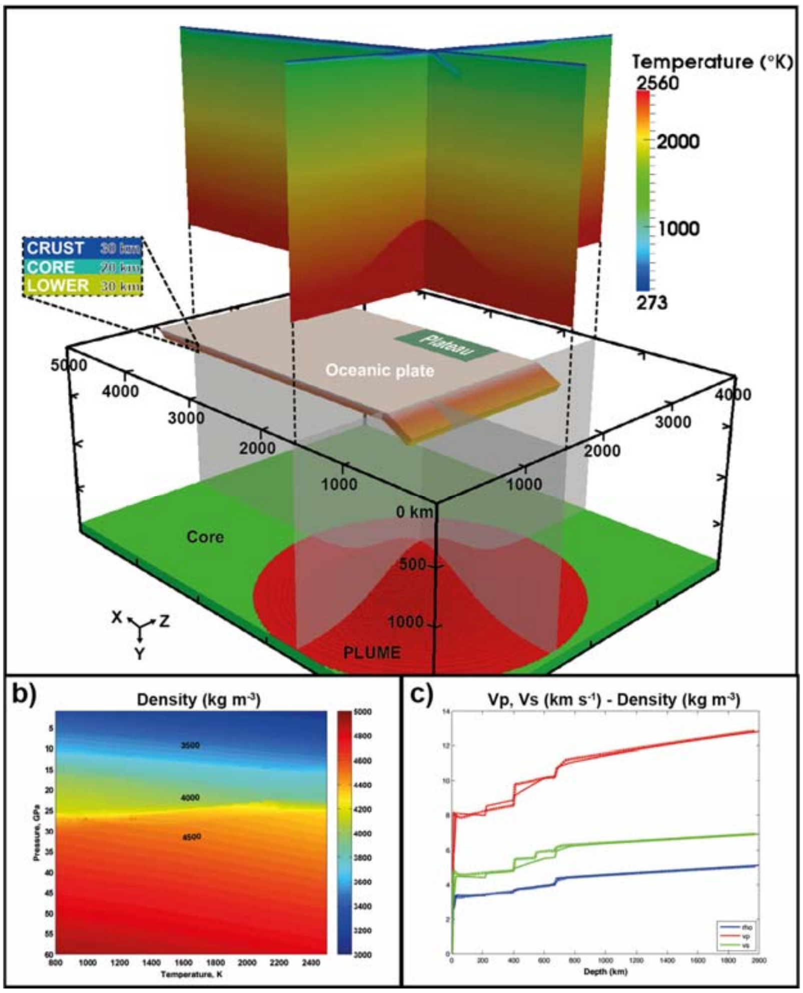

Supplementary Fig. 9:

Initial setup for geodynamic modeling. (**a**) Initial model setup. The oceanic plate is colored according to the depth scale bar in Fig. 4. Left and right side of the plate are defined looking at the direction of subduction. The mantle and background crust surrounding the plate are not visualized. (**b**) Density map generated with PERPLE_X[2] for a pyrolitic mantle composition and zoomed in the P-T range relevant for this study. (**c**) Vertical profiles of density and isotropic Vp and Vs from PREM[3] (continuous lines) and calculated in the geodynamic model along a background mantle geotherm (dashed lines).

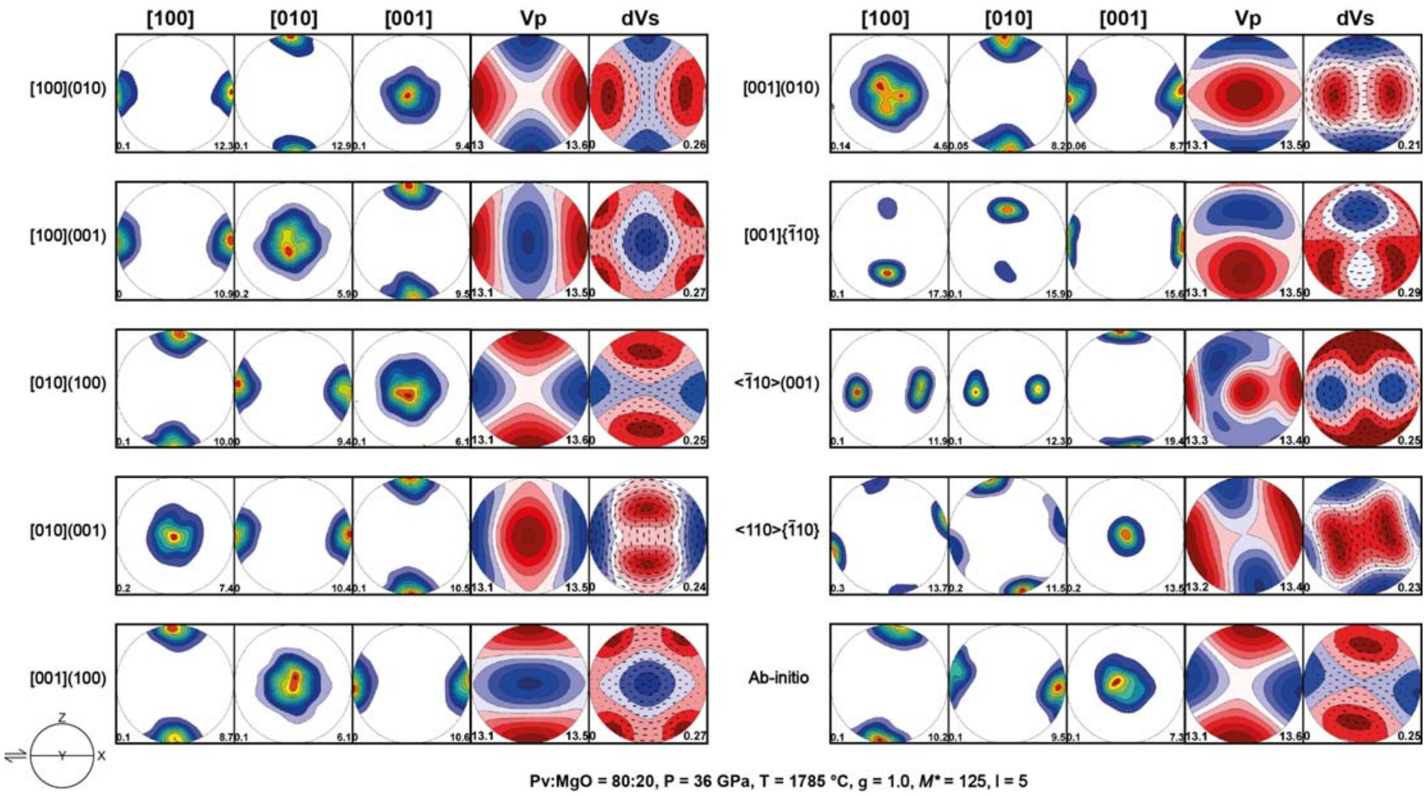

Supplementary Fig. 10:

Bridgmanite LPOs and associated elastic properties during horizontal simple shear deformation (γ=1). For each fabric, we show the pole figures of the orientation of the principal crystallographic axes (red is maximum; blue is minimum), together with Vp (km/s) and dVs (km/s) calculated at lower mantle conditions and for a pyrolitic composition (red is minimum; blue is maximum). Cubic MgO crystals are random. The bars in the dVs maps indicate the polarization of the fast shear wave component for different propagation directions. The fabric on the bottom right was obtained with CRSS at 36 GPa reported in (ref. 4) and indicated in Supplementary Table 2. The other 9 fabrics are characterized by an easy glide system (indicated on the left) in which slip is 5 times easier than slip on all other systems. The D-Rex parameters for dynamic recrystallization (*M**, *λ**) are indicated at the bottom.

Supplementary Table 1:

Thickness and physical properties for the different rock types.

Rock type (dimensions)	Thickness (km)	A_D (Pa^n s^{-1})	n	E (J)	V (J bar^{-1})	C_{DP} (MPa)	μ_0	μ_1	ε_1	τ_{II_trans} (MPa)	ρ (kg m^{-3})	k (W m^{-1} K^{-1})
Background crust	30	$1.97 \cdot 10^{17}$	2.3	$1.54 \cdot 10^5$	1.2	1	0.01	0.01	-	0.03	3200§	$1.18+\frac{474}{T+77}$
Slab crust	30	$1.97 \cdot 10^{17}$	2.3	$1.54 \cdot 10^5$	1.2	1	0.01	0.001	0.05	0.03	TD	$1.18+\frac{474}{T+77}$
Slab mid layer	20	10^{24}	-	-	-	-	-	-	-	-	TD	$0.73+\frac{1293}{T+77}$
Slab lower layer	30	$3 \cdot 10^{22}$	-	-	-	-	-	-	-	-	TD	$0.73+\frac{1293}{T+77}$
Plateau	30	$4.80 \cdot 10^{22}$	3.2	$2.38 \cdot 10^5$	0.0	1	0.15	0.15	-	0.03	2950	$1.18+\frac{474}{T+77}$
Plume	-	$3.98 \cdot 10^{16}$	3.5	$5.32 \cdot 10^5$	1.0	1	0.6	0.6	-	30	TD	$0.73+\frac{1293}{T+77}$
Upper mantle + Transition zone	630	$3.98 \cdot 10^{16}$	3.5	$5.32 \cdot 10^5$	1.2	1	0.6	0.6	-	0.03	TD	$0.73+\frac{1293}{T+77}$
Lower mantle	1340	$3.98 \cdot 10^{16}$	3.5	$5.32 \cdot 10^5$	1.2	1	0.6	0.6	-	30	TD	$0.73+\frac{1293}{T+77}$
Core	100	$1 \cdot 10^{18}$	-	-	-	-	-	-	-	-	10000	$0.73+\frac{1293}{T+77}$

From left to right: **initial thickness** of the given rock type layer; **pre-exponential factor** (where no other rheological parameters are given, indicates the assigned constant viscosity with dimensions of Pa · s); **power-law exponent**; **activation energy**; **activation volume**; **cohesion**; **initial and final friction coefficient**; **amount of strain** to acquire maximum plastic weakening (i.e., final friction coefficient); **transition stress** below which diffusion creep is active; **density** (TD: density calculated according to the thermodynamic database; values indicate assigned constant densities); **thermal conductivity**.

§ If entrained below 80 km depth by the slab, density is calculated according to the thermodynamic database to reflect full eclogitization.

Supplementary Table 2:

Critical resolved shear stresses (CRSS) normalized by the easiest slip system.

Slip system [uvw](hkl)	A-type Olivine[a]	Enstatite[a]	A-type Wadsleyite[b]	Ab-initio Bridgmanite[c]
[100](010)	1	-	1	1.9
[100](001)	2	-	5	4.7
[100](011)	-	-	5	-
[100](021)	-	-	5	-
[010](100)	-	-	-	1
[010](001)	-	-	-	2.7
[001](100)	-	1	-	6.3
[001](010)	3	-	5	8.1
[001]{$\bar{1}$10}	-	-	-	4
<$\bar{1}$10>(001)	-	-	-	4.1
<110>{$\bar{1}$10}	-	-	-	4.3
1/2<111>{101}	-	-	5	-

[a] from ref. 5; olivine LPO obtained by comparison with low water content LPO of ref. 6

[b] obtained by comparison with the low water content LPO of ref. 7

[c] from ref. 4.

Supplementary References

1. Christensen, U.R. The influence of trench migration on slab penetration into the lower mantle. *Earth Planet. Sci. Lett.* **140**, 27-39 (1996).

2. Connolly, J. A. D. Computation of phase equilibria by linear programming: a tool for geodynamic modeling and its application to subduction zone decarbonation. *Earth Planet. Sci. Lett.* **236**, 524-541 (2005).

3. Dziewoński, A.M. & Anderson, D.L. Preliminary reference Earth model. *Phys. Earth Planet. Inter*. **25**, 297-356 (1981).

4. Mainprice, D., Tommasi, A., Ferré, D., Carrez, P., Cordier, P. Predicted glide systems and crystal preferred orientations of polycrystalline silicate Mg-Perovskite at high pressure: Implications for the seismic anisotropy in the lower mantle. *Earth Planet. Sci. Lett.* **271**, 135-144 (2008).

5. Kaminski, E., Ribe, N. M. and Browaeys, J. T. D-Rex, a program for calculation of seismic anisotropy due to crystal lattice prefferred orientation in the convective upper mantle. *Geophys. J. Int.* **158**, 744-752 (2004).

6. Zhang, S. and Karato, S.-H. Lattice preferred orientation of olivine aggregates deformed in simple shear. *Nature* **375**, 774-777 (1995).

7. Demouchy, S., Mainprice, D., Tommasi, A., Couvy, H., Barou, F., Frost, D. J., Cordier, P. Fosterite to wadsleyite phase transformation under shear stress and consequences for the Earth's mantle transition zone. *Phys. Earth Planet. Int.* **184**, 91-104 (2011).
